# Effectiveness of the online Acceptance and Commitment Therapy intervention “Embrace Pain” for cancer survivors with chronic painful chemotherapy-induced peripheral neuropathy: study protocol for a randomized controlled trial

**DOI:** 10.1186/s13063-022-06592-3

**Published:** 2022-08-09

**Authors:** Daniëlle L. van de Graaf, Floortje Mols, Hester R. Trompetter, Marije L. van der Lee, Karlein M. G. Schreurs, Elin Børøsund, Lise Solberg Nes, Tom Smeets

**Affiliations:** 1 CoRPS - Center of Research on Psychological disorders and Somatic Diseases, Department of Medical and Clinical Psychology, Tilburg University, PO Box 90153, Tilburg, 5000 LE The Netherlands; 2 Department of Research, Netherlands Comprehensive Cancer Organisation (IKNL), Utrecht, The Netherlands; 3 Centre for Psycho-Oncology, Scientific Research Department, Helen Dowling Institute, Bilthoven, The Netherlands; 4 Department of Psychology, Health & Technology, Centre for eHealth & Well-being Research, University of Twente, Enschede, The Netherlands; 5 Department of Digital Health Research, Division of Medicine, Oslo University Hospital, Oslo, Norway; 6 Faculty of Health and Social Sciences, University of South-Eastern Norway, Drammen, Norway; 7 Department of Psychiatry and Psychology, College of Medicine and Science, Mayo Clinic, Rochester, MN USA; 8 Institute of Clinical Medicine, Faculty of Medicine, University of Oslo, Oslo, Norway

**Keywords:** CIPN, Cancer survivors, Acceptance and Commitment Therapy, eHealth, Online, PROFILES registry

## Abstract

**Background:**

About 30% of cancer survivors suffer from chemotherapy-induced peripheral neuropathy (CIPN) ≥6 months after completion of chemotherapy. This condition, for which treatment options are scarce, comes with limitations in daily life functioning and decreased quality of life. The current study examines the effectiveness of an online self-help intervention based on Acceptance and Commitment Therapy (ACT) in comparison to a waiting list condition (WLC) to deal with CIPN. In addition, it examines which factors moderate effects and to what extent the effects differ between guided and unguided ACT intervention.

**Methods:**

A two-parallel, non-blinded randomized controlled trial (RCT) will be carried out. Adult cancer survivors who experience painful CIPN for at least 3 months and completed chemotherapy at least 6 months ago will be recruited (*n*=146). In the intervention condition, participants will follow an 8-week self-management course containing 6 modules regarding psychoeducation and ACT processes, including therapeutic email guidance. By means of text and experiential exercises, supplemented with illustrations, metaphors, and audio files, people will learn to carry out value-oriented activities in their daily life with pain. Participants will learn new ways of coping with pain, including reducing pain avoidance and increasing pain acceptance. Participants in the WLC will be invited to follow the intervention without therapeutic guidance 5 months after start. Pain interference is the primary outcome, while psychological distress, quality of life, CIPN symptom severity, pain intensity, psychological flexibility, mindfulness skills, values-based living, and pain catastrophizing will serve as secondary outcomes. All outcome measures will be evaluated at inclusion and baseline, early-intervention, mid-intervention, post-treatment, and 3- and 6-month post-treatment. Qualitative interviews will be conducted post-treatment regarding experiences, usage, usability, content fit, and satisfaction with the intervention.

**Discussion:**

This study will provide valuable information on the effectiveness of an online self-help intervention based on ACT versus WLC for chronic painful CIPN patients.

**Trial registration:**

ClinicalTrials.gov NCT05371158. Registered on May 12, 2022.

Protocol version: version 1, 24-05-2022

**Supplementary Information:**

The online version contains supplementary material available at 10.1186/s13063-022-06592-3.

## Background

The number of cancer survivors continues to grow [1–5] as the population ages, early detection through screening rises, and cancer survival rates improve [6]. Many of these survivors face long-term side effects of cancer and its treatment, whereby their quality of life (QoL) is strongly affected [1, 7]. Chemotherapy can cause multiple disabling long-term side effects like chemotherapy-induced peripheral neuropathy (CIPN) [8]. CIPN can occur due to the use of certain chemotherapeutic agents, such as taxanes, platinum compounds, and vinca alkaloids [9]. CIPN presents itself with symptoms such as tingling, numbness, cramps, and aching or burning pain in the hands and feet, which can also spread to arms and legs [10–12]. Up to 80% of cancer survivors suffers from CIPN 1 month after chemotherapy, which stabilizes to around 30% at 6 months or longer after chemotherapy [7, 8, 13–16]. This prevalence differs between cancer types (range 10–60%). As the application of chemotherapy in cancer treatment is rising, CIPN is likely to become one of the most common long-term side effects for cancer survivors [17]. CIPN is a very limiting condition, as a systematic review has shown that CIPN negatively influences QoL in adult cancer survivors [7]. Patients can experience impaired QoL due to CIPN up to 11 years after chemotherapy, caused by the decreased performance of regular activities, depressive symptoms, and poor sleep quality [18–21]. In addition, it has been shown that specifically *painful* CIPN is associated with lower QoL compared to non-painful CIPN [22].

In order to improve the patients’ QoL, cognitive behavioral therapies (CBT), like third-generation CBTs as Acceptance & Commitment Therapy (ACT), are increasingly used in global cancer care [23, 24]. ACT helps patients to shift focus towards engaging in personally valuable activities by increasing pain acceptance [25] and has been shown to be effective for other types of chronic pain [26]. Although the effectiveness of online ACT interventions has not yet been investigated for chronic painful CIPN patients, a previous study found that treatment with online CBT positively affected pain intensity in this patient group, creating positive expectations for treatment with online ACT as well [27]. Traditional *face-to-face* psychological therapies have some drawbacks, such as high costs, not being accessible or hardly available, having to travel to the therapist, negative stigma, and high psychological burden [27, 28]. Due to developments in the field of eHealth, these obstacles can be mitigated [27–29]. That is, with the use of online self-management interventions, patients are able to receive psychological therapy whenever and wherever they would like to, without having to travel to a therapist [27, 28]. Online self-management interventions offer the additional benefit of augmented protection of patients’ anonymity and privacy [29].

Even though online interventions are relatively new, several such interventions have demonstrable effectivity in improving QoL in cancer patients and survivors [28, 30–32]. A randomized controlled trial (RCT) has shown that the use of an online CBT intervention significantly improved CIPN pain intensity and may be effective [27]. Furthermore, research has shown that online interventions based on ACT are capable of improving pain intensity, pain interference, pain catastrophizing, disability, depression, anxiety, psychological inflexibility, and QoL in chronic pain patients with a variety of pain diagnoses (e.g., fibromyalgia, rheumatoid arthritis, and back complaints) [33, 34]. These findings suggest that an online self-help intervention based on ACT might be effective for adult cancer survivors with chronic painful CIPN as well. However, effects may differ between patients, since previous research has shown factors such as pain intensity, depressive and anxiety symptoms, and emotional well-being to predict the effectiveness of online ACT for chronic pain [35], and the extent to which patients benefit from the online intervention may hence vary. Furthermore, there may also be differences in the effectiveness with different types of the online intervention, namely with or without guidance. Earlier research has shown that guidance, including feedback, explanations, motivations and reminders, improves treatment effects compared to unguided interventions [36, 37]. The primary aim of this study researching the online intervention named ‘Embrace Pain’ is to reduce pain interference in cancer survivors with chronic painful CIPN. To study this, three objectives have been formulated:

- *Objective 1:* To examine the effectiveness of an online self-help intervention based on ACT for reducing pain interference in cancer survivors with chronic painful CIPN in a randomized controlled trial (RCT) and compare this to a waiting list condition (WLC).
- *Objective 2:* To examine which baseline demographic, clinical, and psychosocial factors may moderate the effectiveness of an online self-help intervention based on ACT.
- *Objective 3:* To examine the extent to which effects differ between guided and unguided versions of the online self-help intervention based on ACT.

## Methods

### Study design

**Fig. 1:**
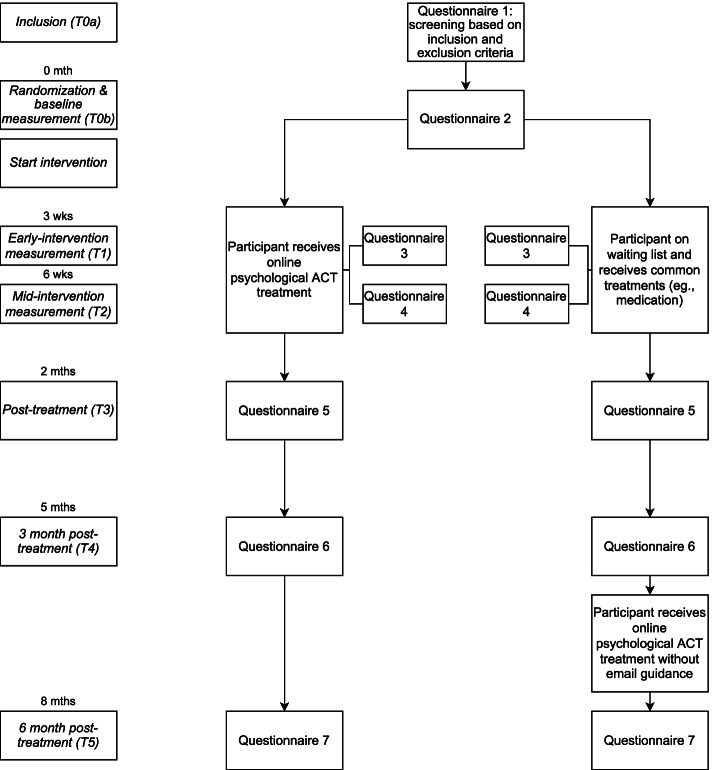
Flowchart

An RCT will be carried out with two parallel, non-blinded groups. Participants will be involved in the study for a duration of 8 months from the first to the last measurement. Outcome measures will be collected at baseline (T0), early-intervention (T1; 3 weeks after baseline), mid-intervention (T2; 6 weeks after baseline), directly at the end of intervention (T3; 2 months after baseline), and through two follow-up measurements (T4; 3 months after the intervention, T5; 6 months after intervention). A flowchart of the study is shown in Fig. 1.

### Study population

Inclusion criteria consist of (a) age of 18 years or older and (b) identified by a clinician or self as having painful sensations (i.e., aching, burning, “pins-and-needles”, shock-like, painful tingling, numbness, cramps) bilaterally in the feet/legs and/or hands/arms for at least 3 months. Furthermore, (c) a score of 3 or higher on an 11-point pain intensity scale (Numeric Rating Scale), (d) the pain was not present prior to receiving chemotherapy, (e) chemotherapy ended at least 6 months ago, and (f) a score of 1 or higher on EORTC QLQ–CIPN20. Exclusion criteria are (a) enrollment in psychological treatment related to cancer, pain, or psychological disorders upon entry; (b) new chemotherapy scheduled during study participation; (c) no access to the Internet/no email address; (d) not enough time to follow the intervention (2 h per week); and (e) according to patient’s own perception no problems with the Dutch language.

### Recruitment

Patients will be recruited through a flyer with a QR code and a link to a website. These flyers will be distributed in various ways (in the Netherlands): online media (e.g., Kanker.nl, Facebook groups for neuropathy, patient associations), hospital waiting rooms (e.g., oncology departments, AYA centers, pain clinics), and through other healthcare providers (e.g., oncological foot care providers). Interested patients can visit the recruitment website where they can read more information and sign up if interested.

When a patient is interested in participation, the patient will receive an information letter and an informed consent form by mail, sent by a research assistant. The informed consent also includes information regarding the collection and use of participant data in follow-up studies. Afterwards, the participant will receive a questionnaire (T0a) regarding inclusion criteria, to examine whether the participant is eligible to participate. Additionally, patients must achieve certain scores on several questionnaires, namely Numeric Rating Scale ≥2 and EORTC QLQ–CIPN20 ≥1. If the questionnaire from T0a shows that respondents are not suitable for participation, they will be notified with a rejection and along with it the reason for rejection based on the relevant inclusion or exclusion criterion.

### Randomization

When the patient is admitted to the study, the patient will receive confirmation of participation, the result of randomization, and the baseline questionnaire (T0b). Patients will be randomized to receive either ACT or WLC. The study will stratify by age and sex by means of block-wise randomization. An online generator will be used for this. Randomization will be performed by a research associate.

### Intervention

The experimental condition includes an online psychological intervention with therapist email guidance based on ACT. The intervention can be worked through by the patient from home or at another location of choice, in a webpage or app by logging in with an email and self-created password. Participants receive a welcome letter from the supervisor. It consists of 6 modules which can be worked through in 8 weeks. The intervention primarily consists of text and experiential exercises, complemented with illustrations, metaphors, and audio (mp3) files. Before starting the module, participants read an introduction about the training. The first module includes psychoeducation on neuropathic pain and CIPN (Table 1). Participants will be provided with information to familiarize themselves with intervention goals and mindfulness exercises central to ACT. In subsequent modules, participants will learn about the aversive effects of pain avoidance, gain insight into their personal values, and work on pain acceptance. Throughout the intervention, participants will exercise to recognize unhelpful thoughts about their pain and learn the difference between the subjective (judging) and objective self, create activities that align with values, and think about concrete actions to prevent relapse. When access to the online environment is terminated, participants can no longer view the online environment or communicate with the supervisor. However, participants can always continue to view the session they submitted.

**Table 1:** Schematic overview of modules of the ACT intervention

Module	Therapeutic processes	Information	Assignments	Audio files (mindfulness)
Welcome		Online environment, contact, additional help, other long-term consequences, and tips.		
1: Chronic neuropathic pain	Psychoeducation	CIPN and chronic pain		A: Concentration on breathing
2: On the way to values	Values	Values and valued-based activities	I: Explore your values II: My values III (optional): My values in pictures	A: Body exploration
3: Away from my values	Pain avoidance	Avoidance and managing pain	I: My ways of avoiding II: Moment when I am in pain	A: Concentration on breathing B: Body exploration C: Three-minute breathing space
4: On the road with skills	Commitment and Defusion	Learning how to deal with pain differently	I: Acceptance in action II: Your recurring thoughts of pain III: Is the thought useful? IX (optional): Struggle or open up	A: Allowing what is
5: Taking a new road	Committed action	Converting values to behaviors in different life areas and devising valued-based activities	I: My ACT matrix	A: Concentration on breathing B: Body exploration C: Three-minute breathing space D: Allowing what is
6: On the road to values: from day to day	Social context	Perform actions in daily life and long-term changes	I: Your communication about pain II: Self-care	A: Three-minute breathing space B: Notice five things C: Brushing your teeth with attention

Participants in the experimental condition will receive guidance from Psychology master’s students from Tilburg University, who will be first trained and then supervised by a licensed healthcare psychologist. In addition, they receive supervision in the first weeks of providing guidance. Also, they will always have the possibility to ask for advice from a licensed clinical psychologist during the intervention. There will also be a backup in case of possible psychological problems that require more help. In that case, the licensed clinical psychologist will be informed and asked for advice. Guidance of participants will occur via email. Contact will be without obligation. It is possible to send an email every week in the protected environment of the online intervention, which complies with all privacy conditions as stated by the GDPR. Guidance is mainly intended for feedback related to the exercises made, answering questions about the content, and maintaining motivation to continue with the intervention. Here the choice lies with the participant, but facilitators will continue to encourage questions throughout the training.

### Waiting list condition

The control condition includes a WLC. At 5 months after start (i.e., T4) participants in the WLC receive the opportunity to follow the intervention without email guidance by a therapist. They neither receive a welcome letter at the start, which means that they only read the introduction about the training before starting.

### Outcomes

Questionnaires will be completed online via the PROFILES Registry [38]. Patients will receive reminders via email to promote participant retention and complete follow-up. A complete overview of enrolment, interventions, and outcome measurements is shown in Table 2.

**Table 2:**
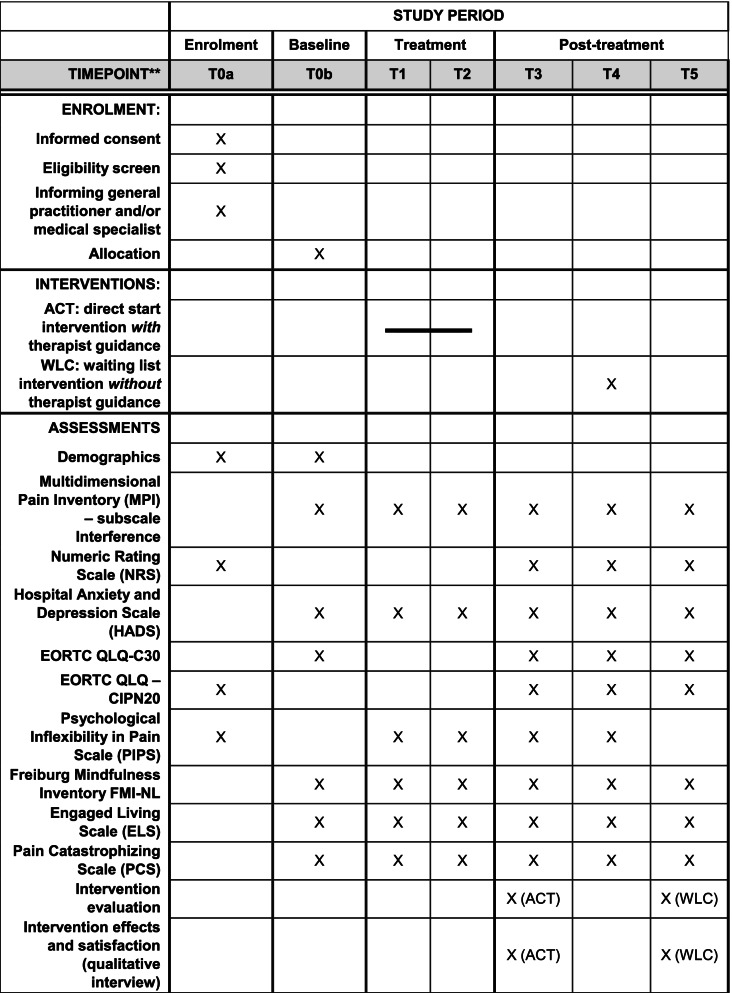
Schedule of enrolment, interventions, and assessments

#### Primary outcome

*Pain interference* will be measured using the Multidimensional Pain Inventory, Dutch language version (MPI-DLV) – subscale Interference [39, 40]. The MPI consists of statements rated on a 7-point Likert scale ranging from 0 (no change) to 6 (a lot of change). The subscale Interference focuses on psychosocial aspects of chronic pain, such as functioning in work, homework, and recreational and social activities. The scale consists of 11 items and has been translated/validated into Dutch [39].

#### Secondary outcomes

*Quality of life* will be measured with the European Organization for Research and Treatment of Cancer Quality of Life Questionnaires Core-30 item (EORTC QLQ-C30) [41]. This is a validated 30-item self-report screening scale for QoL in cancer patients including a 4-point Likert scale ranging from 1 (not at all) to 4 (very much) including five subscales (i.e., physical functioning, role functioning, emotional functioning, cognitive functioning, and social functioning) [41].

*CIPN symptom severity* will be measured using the European Organization for Research and Treatment of Cancer Quality of Life Questionnaire-CIPN20 (EORTC QLQ–CIPN20) [42]. This questionnaire is intended to be used as a supplement to the EORTC QLQ-C30. It assesses CIPN-related symptoms as well as functional limitations related to CIPN. The questionnaire has been validated and includes 20 items on a 4-point Likert scale ranging from 1 (not at all) to 4 (very much) including three subscales (i.e., sensory, motor, and autonomic symptoms) [43].

*Pain intensity* will be measured with the Numeric Rating Scale (NRS-11) [44]. This is based on International Association for the Study of Pain (IASP) recommendations for measures in clinical trials on chronic pain [44]. This questionnaire has been validated and includes 2 items on a 11-point Likert scale ranging from 0 (no pain) to 10 (the worst pain possible) [45].

*Psychological distress* will be measured using the Hospital Anxiety and Depression Scale (HADS) [46, 47]. This is based on IASP recommendations for measures in clinical trials on chronic pain [44]. The HADS is a validated 14-item self-report screening scale (ranging from 0 to 3) including two subscales (i.e., anxiety and depression symptoms) [46, 47].

*Pain catastrophizing* will be measured using the Pain Catastrophizing Scale (PCS) [48]. This questionnaire includes three subscales (i.e., rumination, magnification, and helplessness). It consists of 13 items on a 5-point Likert scale ranging from 0 (not at all) to 4 (always) and has been validated [48].

*Psychological flexibility* will be measured with the Psychological Inflexibility in Pain Scale (PIPS) [49, 50]. This is a validated 12-item measurement, including two subscales (i.e., avoidance and cognitive fusion) [51]. It consists of a 7-point Likert scale, ranging from 0 (never true) to 6 (always true).

*Mindfulness* will be measured using the Freiburg Mindfulness Inventory (FMI) [52]. This validated measurement consists of 14 items with two subscales (i.e., presence and acceptance) [52]. It includes a 4-point Likert scale, ranging from 0 (rarely) to 3 (almost always).

*Values-based living* will be measured with the Engaged Living Scale (ELS) [53]. This is a validated 16-item measurement, consisting of two subscales (i.e., valued living and life fulfillment) [53]. It includes a 5-point Likert scale, ranging from 0 (completely disagree) to 4 (completely agree).

### Intervention evaluation

*Intervention evaluation* includes 15 questions regarding the evaluation of the intervention. It includes questions regarding amount of use and overall satisfaction with the content and guidance, which was based on an earlier RCT studying an online ACT intervention [33]. Overall satisfaction will be measured using the Client Satisfaction Questionnaire (CSQ-8) [54]. Also, participants will grade the intervention on a scale from 1 (extremely poor) to 10 (excellent).

#### Process outcomes

Technical data regarding usage of the intervention will be gathered, namely moment when session was first viewed, moment when session was finished, moment of message sent to supervisor, and moment of feedback given by supervisor. This enables evaluation of adherence to the intervention and information about the use of guidance. Once all participants have completed the online training, Karify will make this data available. Karify is the eHealth platform in which the online intervention was build (ISO 27001 and NEN 7510 certified).

#### Other outcomes

Qualitative data on intervention experiences and satisfaction will be collected by means of interviews with some participants after completion of the intervention. In this way, it will be possible to examine experiences, usage, usability, content fit of intervention with complaints, and satisfaction. Specifically, both participants who adhered and did not adhere to the intervention will be interviewed. Patients adhered to the intervention if all sessions were completed (i.e., based on data retrieved from Karify) and a minimum of 2 h per week was spent on the intervention (i.e., self-reported). Participants are informed about this by the information letter and informed consent form. As a starting point, 4 patients in each group (i.e., adherent and non-adherent) will be interviewed. New interviews will be done until saturation is reached.

#### Participant characteristics

Socio-demographic factors are assessed, including sex, marital status, having children, educational level, and work. Clinical information will be examined as well, including year of diagnosis, tumor type, other cancer treatments besides chemotherapy, CIPN characteristics (i.e., days per week, medication used to treat CIPN), long-term consequences of cancer (other than CIPN), and psychological problems. Comorbidities in the last 12 months will be examined using the Self-administered Comorbidity Questionnaire (SCQ) [55].

### Sample size calculation

Per group, 51 participants during follow-up measurements are necessary to detect minimal effect sizes of interest (*d* = .50) on the primary outcome. This is based on earlier findings on effectiveness of online and face-to-face ACT intervention for chronic pain [33, 34]. The power will be high enough (*p* = 1-beta = .80) to find significant effects in a two-sided test at alpha = .05. As found in previous online ACT interventions, a drop-out rate of 30% needs to be considered. G*Power calculations revealed that 73 participants per group are needed at baseline, which means that 146 participants are needed in total.

### Statistical analysis

#### Primary analyses

Significant differences at baseline between the conditions will be checked by performing one-way ANOVA’s and chi-square tests.

Effects of the intervention on all outcomes will be assessed by performing intention-to-treat analyses using general linear mixed models. Baseline to post-intervention and 3- and 6-month follow-up differences will be used as repeated measures, with treatment (2 levels), time (3 levels) and their first-order interactions as fixed factors. Confidence intervals of 95% will be calculated for all outcomes. Drop-out and non-adherence are relatively common in online interventions [56]. Therefore, secondary analyses will be performed to examine effects on outcome variables for participants who adhered to the intervention (i.e., all session completed and self-reported minimum of 2 h per week spent on intervention). At post-intervention and follow-up, effect sizes (Cohen’s *d*) will be calculated using means and standard deviations. Effect sizes of 0.80 are considered large, effect sizes of 0.50 are evaluated as moderate, and effect sizes of 0.20 are considered small [57].

Initiative on Methods, Measurement, and Pain Assessment in Clinical Trials (IMMPACT) recommendations will be followed to evaluate the proportion of participants showing clinically significant change [58]. Clinical improvement will be assessed with missing values imputed by the Expectation Maximization-algorithm. Proportions of clinically improved participants and Chi-square tests will assess statistically significant differences in the proportions for the two conditions. It will be calculated for (a) pain interference (MPI – Interference), (b) pain intensity (NRS), and (c) CIPN symptom severity (EORTC QLQ – CIPN 20). A decrease of at least 0.6 standard deviation will be assessed as evidence of clinically significant change for the MPI – Interference scale, based on the IMMPACT guidelines [58]. Decreases of 20% or higher in pain intensity (NRS) will be assessed as minimum and moderate changes. No specific IMMPACT guidelines exist for the HADS. Therefore, a distribution-based decrease of at least 1 standard error of the mean is recommended [58].

#### Secondary analyses

To assess predictors of effects, steps of a study on predictors of change during CBT for chronic pain will be followed [59]. Pain interference at 3-month follow-up will be used as indicator of treatment effect. Using the PROCESS macro for SPSS [60], linear regression models will be applied (two-tailed). Predictor variables may include sex, age, educational level, number of comorbidities, time since CIPN onset, CIPN symptom severity (EORTC QLQ – CIPN20), quality of life (EORTC QLQ-C30), pain intensity (NRS), psychological distress (HADS), psychological inflexibility (PIPS), mindfulness (FMI-NL), valued based living (ELS), and pain catastrophizing (PCS). They will be selected based on theoretical and empirical considerations.

To assess differences in effects between the guided and unguided version of the intervention, independent samples t-tests and a two-stage hierarchical multiple regression analysis for each primary and secondary outcome will be conducted. Baseline to 3-month follow-up change will be used as the dependent variable. The independent variable will be an intervention dummy variable. Baseline score on the respective outcome measure will be used as control variable. Effect sizes for both conditions will be computed to indicate differences in the magnitude of the effects of online ACT with and without email counseling.

#### Interview analyses

Qualitative data will be used to gain further insight in the intervention effects, usage, usability, content fit of intervention with complaints, and satisfaction. All interviews will be recorded and transcribed verbatim. Thematic analysis in Atlas Ti 8.0 will be used to analyze the data. Following transcription, two researchers will independently (re)read a set of 3–4 interviews and perform inductive coding followed by a discussion of disparities. One researcher will further code the rest of the interviews using a codebook that will be generated from the first round of coding, adding new codes along the way, and discussing outcomes of coding with the second researcher.

### Ethical considerations

This study protocol has been reviewed and approved by the Medical Research Ethics Committee Brabant, the Netherlands (reference number: NL78436.028.21). If there are protocol modifications, all relevant parties will be informed. Tilburg University has insurance for participants for compensation in the unlikely event that participants are harmed from trial participation.

### Data security

Each participant will be assigned a study number, which guarantees confidentiality and anonymity. Only the research assistant will be able to connect the study numbers to the participants. Data will be stored in a secure location (PROFILES registry) for 15 years. PROFILES data is freely available (FAIR principles) for non-commercial scientific research trough www.profilesregistry.nl [38].

### Output

Trial results will be published in (inter)national peer reviewed scientific journals and will be communicated to the stakeholder group, the Dutch Cancer Society, and Netherlands Comprehensive Cancer Organisation (IKNL). Presentations will be held at (inter)national conferences.

## Discussion

Chronic painful CIPN is a very limiting long-term consequence of chemotherapy that many adult cancer survivors suffer from, resulting in a greatly reduced QoL. Since treatment options are limited, online self-help therapy could offer support to these patients. This study aims to evaluate effectiveness of an online self-help intervention based on ACT. It could provide an entry point for the development of psychological treatment for cancer patients suffering from other forms of cancer pain, which is a major, growing cancer survivorship issue that is highly under recognized.

## Trial status

Recruitment started in December 2021. Estimated Primary Completion Date is July 1, 2023.

## Supplementary Information

**Additional file 1:** Subject information for participation in medicalscientific research.

**Additional file 2: Appendix E.** All items from the World Health Organization Trial Registration Data Set.

## Data Availability

The research team will have access to the final trial coded dataset. They can access it at any time and there is nothing contractually preventing that. The research assistant will have access to all data, both coded and uncoded.

## Abbreviations

ACT: Acceptance and Commitment Therapy
CBT: Cognitive Behavioral Therapy
CIPN: Chemotherapy-induced peripheral neuropathy
PROFILES Registry: Patient Reported Outcomes Following Initial treatment and Long-term Evaluation of Survivorship Registry
RCT: Randomized controlled trial
WLC: Waiting list condition
