# Supplementary material for: Effectiveness of the online Acceptance and Commitment Therapy intervention “Embrace Pain” for cancer survivors with chronic painful chemotherapy-induced peripheral neuropathy: study protocol for a randomized controlled trial
Source: Trials. 2022 Aug 9;23:642. doi: 10.1186/s13063-022-06592-3 (PMC9361507; doi:10.1186/s13063-022-06592-3)
Supplement: Supplementary file 1 — Additional file 1: Subject information for participation in medicalscientific research. [file 13063_2022_6592_MOESM1_ESM.pdf]

Subject information for participation in medical-scientific research

Online training ‘Embrace Pain’

Official title: QLIPP-CIPN: Effectiveness of online Acceptance and Commitment Therapy for improving pain interference in cancer survivors with chronic pain from chemotherapy-induced neuropathy (CIPN)

Introduction

Dear Sir/Madam,

With this information letter we would like to ask you if you would like to participate in a medical-scientific study. In this study we will investigate the effect of an online psychological training. This training has been developed for people who suffer from chronic pain due to nerve damage after chemotherapy. Goal of the training is to reduce the limitations caused by the pain in daily life. Participation is voluntary.

You can read here what kind of research it involves, what it means for you, and what the advantages and disadvantages are. It is a lot of information. Please read the information and decide if you want to participate. If you want to participate, you can sign up at https://www.profielstudie.nl/qlipp-cipn-neuropathie-project-voor-mogelijke-deelnemers/

Ask your questions

You can make your decision with the information found in this information letter. In addition, we encourage you to:
- Ask questions of the researcher who is giving you this information. You can find the details at the bottom of this letter.
- Talk to your partner, family or friends about this study.

You can also ask questions of and independent expert. You can find the details at the bottom of this letter.

1. General information

This research was designed by Tilburg University. Researchers from Tilburg University are conducting this research. The Brabant Medical Ethics Review Committee has approved this research.

2. What is the background of this study?

One of the side effects after chemotherapy is pain due to nerve damage, or peripheral neuropathy. In full, this nerve damage is called "chemotherapy induced peripheral neuropathy" (CIPN). The pain often occurs on two sides of the body in the feet and/or hands. It may spread to the legs and/or arms. The pain may feel burning, stabbing, numb or tingling

and may be accompanied by cramping, muscle weakness or other symptoms. Unfortunately, the pain after CIPN persists over the long term in a portion of people.

For many people who continue to suffer from long-term pain - due to CIPN or other causes - medical solutions do not help enough (anymore). They may be told that they have to learn to live with the pain. This is often a difficult message. In this research we are developing a new method to help people continue to live with pain due to CIPN. The method is opposite to what people often do in response to pain. This method focuses not so much on the pain symptoms themselves, but on the valuable things in life. People learn to accept the pain, but especially negative feelings and thoughts.

3. What is the aim of this study?

Tilburg University wants to investigate whether shifting the focus to a valuable life leads to less pain in daily life. The research is not directly aimed at taking away the pain itself. Often this is not possible anymore. However, the intensity of the pain can decrease by learning to pay more attention to a valuable life with pain. In the study we compare people who follow an online training with a group of people who are temporarily on a waiting list for this training.

4. How does the study proceed?

How long does the study last?
The study takes a total of 3 to 6 months. The training itself lasts 8 weeks. After the training we will ask you to complete a few more questionnaires.

Step 1: Register
If you are interested in the study, you can register on the website https://www.profielstudie.nl/qlipp-cipn-neuropathie-project-voor-mogelijke-deelnemers/. You can read more information about the study on the website. There you can also read which criteria you have to meet to be able to participate. For example, you have to have been suffering from pain caused by chemotherapy for more than three months, not have received chemotherapy in the past six months, be 18 years of age or older, have enough time to participate in the study, and have access to the Internet at home. In addition, you cannot participate if new chemotherapies are scheduled during the study.

If you decide to participate in the study, you can register on the website. The researchers will send you a consent form by mail. You will then have 1 week to think calmly about whether you want to participate in the study. If you wish to participate, sign the consent form and return it to the researchers via mail.

When you consent to participate in the study, we will also test some of the criteria that you must meet. You will therefore complete a questionnaire. You will receive an invitation to do so by email after the researchers have received your consent form. Once you have completed

the screening, we will determine whether you meet the requirements and can therefore actually participate in the study.

If you can participate in the study, you will receive an e-mail. This will contain a link to a second questionnaire. You will be asked to complete the questionnaire within a few days.

Step 2: online training

For this study, we will make 2 groups:

- Group 1. The people in this group will receive the online training "Embrace Pain".
- Group 2. The people in this group will be put on a waiting list and follow the online training 'Embrace Pain' 6 months later, without the guidance of a therapist.

After receiving your completed questionnaire, the draw will take place. You will receive the result of the draw by email: either you follow the online training 'Embrace Pain', or you will temporarily be put on a waiting list. If you are allowed to follow the online training, you will receive a link to start the online training.

Each participant has an equal chance to be in a certain group. We cannot change the outcome of the draw. This means that you have no say in which group you will be placed. It is important to realize that in both groups you will eventually follow the online training: in one case immediately and in the other case after 6 months on the waiting list. Thus, you will always follow the training.

<u>Training ‘Embrace Pain’</u>

The training focuses on your thoughts, feelings and reactions to your pain. You will learn to pay more attention to the here and now, to live more from what you consider important and to accept negative feelings and thoughts about your pain. You will learn this by means of examples and short meditation and other exercises. The training is based on Acceptance and Commitment Therapy (ACT).

The training is entirely online. This means that every week you go through the text and exercises on the internet, independently. In total this will take you about 2 hours per week. You can plan when you work on the training during the week. This can also be spread over several moments. The training consists of 6 parts, each week a new part is covered. In total you can take 8 weeks to complete the training.

During these 8 weeks you will receive weekly feedback by email from a supervisor. The guidance is given by specially trained persons who will work under the supervision of a GZ-psychologist. The supervisor who gives you feedback will always be the same person.

Step 3: investigations and measurements

<u>Questionnaires</u>

During the study, we will use online questionnaires. It is important that both participants of the online training and people on a waiting list fill out the questionnaires. The questionnaires are about the extent to which the pain hinders you in your daily life, the extent to which you do what you really find important in your life, and the intensity of your pain and other symptoms of CIPN. Completion of these questionnaires takes place at 7 different times: during the screening, when the study starts, during the study (twice), immediately after the study ends, and three and six months after the study ends. The screening takes about 10 minutes to complete. Completing the questionnaires before and after the study takes about 45-60 minutes each time. Completing the questionnaires during the study takes about 20 minutes. In the appendix you will find a schematic overview of the steps to be followed during the examination.

<u>Interviews</u>
During the study, you may be asked if you are interested in participating in an interview after the study is completed. We will therefore ask for your permission to do so separately. The purpose of this interview is to gain more insight into your progress during the training and your ideas about positive or negative points of the training.

In appendix C you will find a schematic overview of the study.

5. What side effects, adverse reactions or discomforts may you experience?

There are not expected to be any risks associated with participation. You can stop the study at any time.

You do need to invest time to follow the intervention. It takes about 2 hours per week. In addition, it can be confronting to work on your neuropathic pain.

6. What are the advantages and disadvantages of participating in the study?

Participating in the study can have advantages and disadvantages. Below we list them. Think about these carefully, and talk about them with others.

Advantages:

- You will be offered free online training. Even if you end up on a waiting list, you will still have the opportunity to take a training course after the study ends (six months after the draw).
- We expect that the online training will help you better deal with your pain symptoms and that the pain will hinder you less in your daily life.
- With the help of the online training you can (again) realize more valuable aspects in your life and get more grip on your life with pain complaints.

- You are making a significant contribution to understanding what works for neuropathic pain.

Disadvantages:

- If you end up on a waiting list, you will not be able to attend training until 6 months have passed.
- Participation in the study entails completing a questionnaire at 7 points.
- Participation also entails that you have to spend time on the training every week. On average this is 2 hours per week.
- It is possible that your problems will get worse before they get better. This is not because the pain itself is getting worse, but because you are more aware of the pain by being active with it.
- It can be confronting to work on accepting your pain symptoms.

7. When does the study stop?

The researcher will let you know if there is new information about the study that is important to you. The researcher will then ask you if you want to continue participating.

In these situations, the study stops for you:

- The end of the entire study has been reached.
- You want to stop the study yourself. This is possible at any time. Report this immediately to the researcher. You do not have to tell the investigator why you are stopping.

What happens if you stop the study?
The researchers will use the data collected up until the moment you stop.

The entire study ends when all participants have finished.

8. What happens after the study?

Will you receive the results of the study?
The results of the study will be announced through the PROFILE study website (www.profielstudie.nl).

9. What do we do with your data?

Are you participating in the study? If so, you also give us permission to collect, use and store your data.

What data do we keep?
We keep these data:
- your name
- your gender

- your email address
- your age
- data about your health
- (Medical) data we collect during the study

How do we protect your privacy?

All data collected in this study will be kept confidential and encrypted. This means that your data will be given a code that cannot be traced back to you. All completed questionnaires will be kept separate from your personal data. This will leave only anonymous research data. During the course of the study, you can always contact the researchers if you would like to see your data.

Who can see your data?

Some people may have access to all your data. Also to the data without a code. This is necessary in order to check whether the research has been carried out properly and reliably. Persons who are allowed to inspect your data are employees of the Health Care Inspectorate. They will keep your data confidential. The survey will be conducted in the Profile system. In order to fix technical problems in this system, employees of CentERdata can get access to your personal data. CentERdata will remove your personal data immediately after the check or support. We ask you for your permission to do so.

How long do we keep your data?

Your data must be kept for 15 years.

Your data may also be important for other scientific research in the field of cancer after the end of this study. You can indicate on the consent form whether or not you agree to this. If you do not agree, you can simply participate in the current study.

May we use your data for other research?

It is also possible that a follow-up study will take place in the future. In that case we would like to contact you again. You can indicate on the consent form whether or not you wish to be approached for this. If you do not agree, you can simply participate in the current study.

Can you withdraw your permission for the use of your data?

You can always withdraw your permission for the use of your personal data. This applies to this study as well as to the retention and use for any future research. Research data collected up to the time you withdraw your consent will still be used in the research.

We send your data outside the European Union

In this research it is possible that your encrypted data will also be sent to countries outside the EU. Within the PROFILE registration (anonymized) data is shared with other researchers worldwide to obtain new insights. In those countries the EU rules for the protection of your personal data do not apply. However, your privacy will be protected at an equivalent level. If

you do not want your data to be shared with countries outside the EU, you cannot participate in the study.

Would you like to know more about your privacy?

- Would you like to know more about your rights when processing personal data? If so, please visit www.autoriteitpersoonsgegevens.nl.
- Do you have questions about your rights? You can read about your rights on the Tilburg University website https://www.tilburguniversity.edu/disclaimer/privacy-statement/.
- If you have complaints about the processing of your personal data, we recommend that you first discuss them with the research team. You can also go to the Data Protection Officer of Tilburg University. Or you can file a complaint with the Dutch Data Protection Authority.

10. Will you receive compensation for participating in the study?

The online training for the study will cost you nothing. You will also receive no compensation if you participate in this study.

11. Are you insured during the study?

Insurance has been provided for everyone who participates in this study. The insurance pays for damage caused by the research. But not for all damages. In Appendix B you will find more information about the insurance and the exceptions. It also tells you who you can report damage.

12. You can inform your general practitioner or treating specialist.

The researchers have prepared a general letter explaining the study. You can give this to your general practitioner and/or treating specialist if you wish. This is not mandatory.

13. Do you have any questions?

If you would like more information after reading this letter, you can always contact the person conducting the study. This can be done at Mrs. Daniëlle van de Graaf (email: omarm-pijn@tilburguniversity.edu, telephone number: 013 466 4633). During or after the study you can also ask questions and obtain information from the researcher.

Would you like independent advice about participating in the study? Then you can contact an independent physician: (1) Mr. Cornelis Goedhart (physician), tel: 06 22 99 47 80 (telephone: Monday to Thursday) or (2) Peter Kunst (medical advisor and lung specialist): tel: 06 52 41 82 66 (telephone: Monday).

14. How do you consent to the study?

If you decide to participate, we will ask for your consent to participate in the study. This can be done by signing and sending in a consent form. With this you confirm your intention to

participate in the study. It also confirms that we have informed you sufficiently about the study. Even after granting permission to participate, you are free to stop at any time. However, the data collected from you will be stored and used until you stop.

Thank you for your time.

16. Attachments tot his information

A. Contact information

B. Insurance information

C. Schematic overview of the steps to be taken in the study

D. Consent form subject

Appendix A: contact information of Tilburg University

Researcher
Name: Daniëlle van de Graaf
Email: omarm-pijn@tilburguniversity.edu
Phone number: 013 466 4633
Availability: Monday to Friday

Independent physician (1)
Name: mr. drs. Cornelis Goedhart
Function: physician
Phone number: 06 22 99 47 80
Availability: Monday through Thursday

Independent physician (2)
Name: Peter Kunst Peter Kunst
Function: medical advisor and lung specialist
Phone number: 06 52 41 82 66
Availability: Monday

Data Protection Officer of Tilburg University
Name: Mr. Moswa Herregodts
Phone number: 013 466 3359
Availability: Monday through Friday

Data Protection Officer of PROFILES
Email: fg@iknl.nl

Appendix B: insurance information

Tilburg University has taken out a subject insurance policy with Marsh. The maximum insured amount is 650,000 per subject with a maximum of 5,000,000 per scientific research. Any damage can be reported to the researcher.

Appendix C: Schematic overview of the steps to be taken in the study

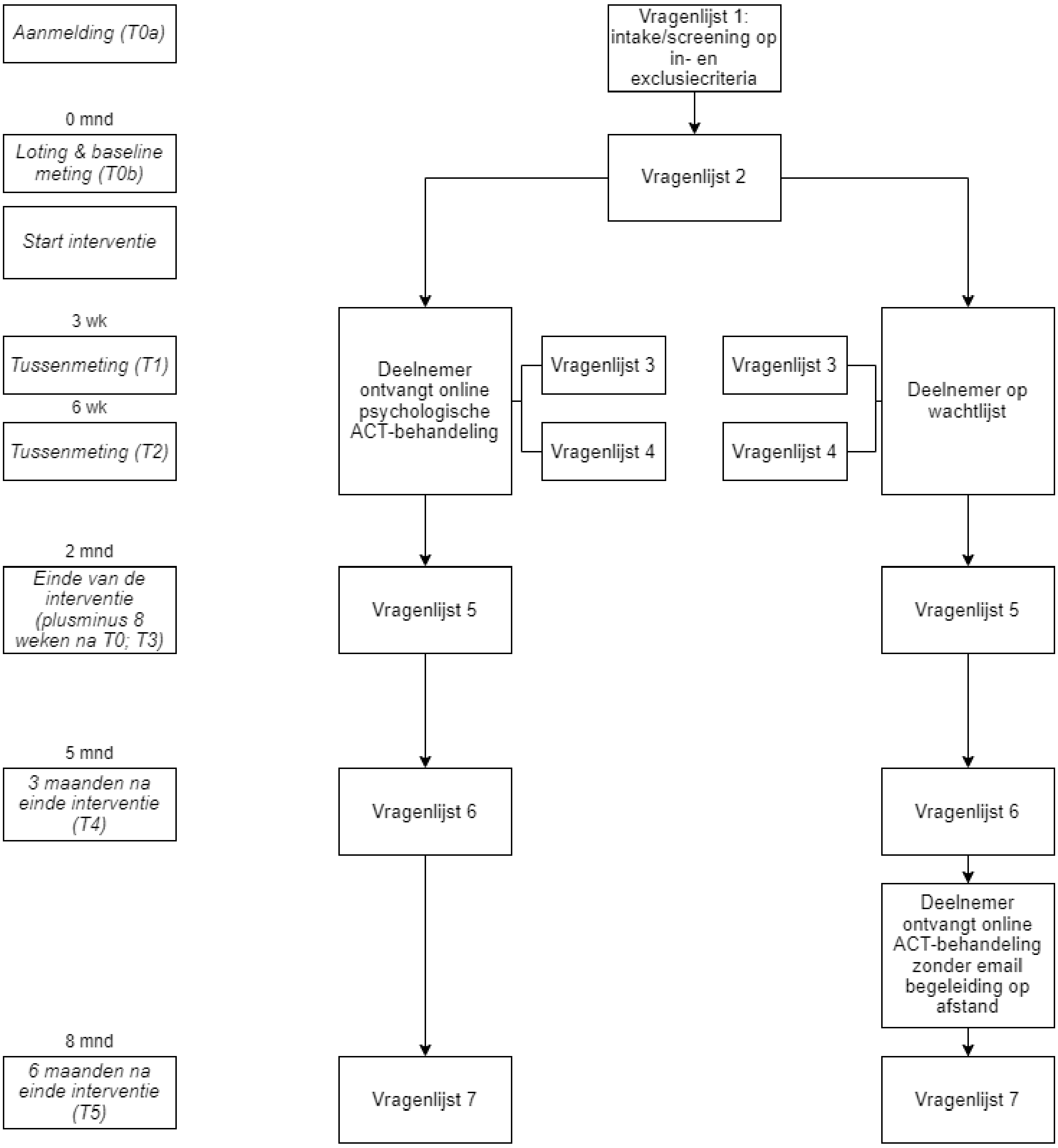

Appendix D: Consent form subject

Study number: | | | | | | | | |

CONSENT FORM

For participation in the scientific study: 'Embrace Pain'

By signing this form, I certify the following:

- I have been satisfactorily informed about the study.
- I have read the written information carefully. I was able to ask additional questions. My questions were adequately answered. I had enough time to decide whether to participate.
- I know that participating is voluntary. I also know that I can decide at any time not to participate or to stop the study. I do not have to give a reason for doing so.
- I consent to the collection and use of my data, including special personal data as further explained in the written information, for the purposes stated in the written information.
- I know that my data will be used for (further) scientific research and made available to researchers for analysis, without this data being traceable to me personally.
- I consent to the sharing of my coded data with other parties in the context of scientific research.
- I am aware that my data can also be made available to employees of the Health Care Inspectorate, should the scientific research be audited.
- The study is conducted in the system Profile. In order to solve technical problems in this system, employees of CentERdata can have access to your personal data. CentERdata will delete your personal data immediately after the check or support.
- I give permission to keep my data at least 15 years after the end of this research.
- I know that my encrypted data can be sent to countries outside the EU where EU privacy rules do not apply.

Cross out what does not apply

- I **do / do not** give permission for the re-use of data for (further) studies in this research field
- I **do / do not** give permission to approach me again for a possible follow-up study after this research.

Your physician/specialist

You have the option of having your primary care physician/specialist informed by the researchers. Your GP/specialist will then be informed that you are participating in this study. You can choose whether you want this or not. This will not affect your participation in this study. In either case, you can sign up for this study.

Cross out what does not apply

I **do / do not** want my primary care physician/specialist to know that I am participating in this study.

I **do / do not** give the researchers permission to inform my GP/specialist.

If chosen "do" in both statements, complete the information below.

GP/specialist name: ....

(If specialist) name hospital: ....

(If specialist) department of hospital: ....

Street name/P.O. Box: ....

Postal code: ....

City: ....

Signing

I want to participate in this study.

Patient Signature: ....

Date: ....

Email address: ....@.....................................

Signature researcher

The undersigned hereby declares that the above person has been informed about the study. If any information becomes known during the study that could affect the person's consent, he/she will inform the person in a timely manner.

Investigator name: ....
(or representative)

Signature: ....

Date: ....
