# Supplementary material for: Effectiveness of the online Acceptance and Commitment Therapy intervention “Embrace Pain” for cancer survivors with chronic painful chemotherapy-induced peripheral neuropathy: study protocol for a randomized controlled trial
Source: Trials. 2022 Aug 9;23:642. doi: 10.1186/s13063-022-06592-3 (PMC9361507; doi:10.1186/s13063-022-06592-3)
Supplement: Supplementary file 2 — Additional file 2: Appendix E. All items from the World Health Organization Trial Registration Data Set. [file 13063_2022_6592_MOESM2_ESM.docx]

Appendix E. All items from the World Health Organization Trial Registration Data Set

| **Data category** | **Information** |
| --- | --- |
| Primary registry and trial identifying number | ClinicalTrials.gov NCT05371158 |
| Date of registration in primary registry | May 12, 2022 |
| Secondary identifying numbers | N.A. |
| Source(s) of monetary or material support | Dutch Cancer Society |
| Primary sponsor | Dutch Cancer Society |
| Secondary sponsor(s) | Tilburg University  Comprehensive Cancer Centre The Netherlands |
| Contact for public queries | Daniëlle L. van de Graaf, MSc  +31134664633  d.l.vdgraaf@tilburguniversity.edu |
| Contact for scientific queries | Daniëlle L. van de Graaf, MSc  +31134664633  d.l.vdgraaf@tilburguniversity.edu |
| Public title | Effectiveness of Online ACT for Pain Interference in Cancer Survivors With Chronic Painful CIPN (QLIPP-CIPN) |
| Scientific title | Patient-centered Development and Effectiveness of Online Acceptance and Commitment Therapy for Pain Interference in Cancer Survivors With Persistent Painful Chemotherapy-induced Neuropathy |
| Countries of recruitment | The Netherlands and Dutch speaking areas in Flanders |
| Health condition(s) or problem(s) studied | CIPN |
| Intervention(s) | Online psychological intervention with therapist email guidance based on Acceptance & Commitment Therapy |
| Key inclusion and exclusion criteria | Inclusion criteria: age of 18 years or older, identified by a clinician or self as having painful sensations (i.e., aching, burning, ''pins-and-needles'', shock-like, painful tingling, numbness, cramps) bilaterally in the feet/legs and/or hands/arms for at least 3 months, score a 3 or higher on an 11-point pain intensity scale (Numeric Rating Scale), the pain was not present prior to receiving chemotherapy, chemotherapy ended at least 6 months ago.  Exclusion criteria: enrollment in psychological treatment related to cancer, pain, or psychiatry upon entry, new chemotherapy scheduled during study participation, no access to the Internet/no email address, not enough time to follow the intervention (2 hours per week), problems with the Dutch language. |
| Study type | Interventional Allocation: randomized Intervention model: parallel assignment  Masking: none (Open Label) Primary purpose: treatment Phase: not applicable |
| Date of first enrolment | February 2022 |
| Target sample size | 146 |
| Recruitment status | Recruiting |
| Primary outcome(s) | Pain interference |
| Key secondary outcomes | Cancer related quality of life, CIPN symptom severity, pain intensity, pain catastrophizing, psychological distress, psychological flexibility, mindfulness, values-based living, intervention evaluation, adherence, interviews. |
